# Influence of Lens Systematic Errors on Autocollimator Angle Measurement: Theoretical and Experimental Explanations

**DOI:** 10.3390/s25247654

**Published:** 2025-12-17

**Authors:** Yuechao Li, Shuo Zhang, Di Chang, Tongmiao Yu, Jiubin Tan

**Affiliations:** 1 Center of Ultra-Precision Optoelectronic Instrument Engineering, Harbin Institute of Technology, Harbin 150080, China; 22b901022@stu.hit.edu.cn (Y.L.); jbtan@hit.edu.cn (J.T.); 2 Key Lab of Ultra-Precision Intelligent Instrumentation, Ministry of Industry and Information Technology, Harbin Institute of Technology, Harbin 150080, China; 3 School of Future Technology, Harbin Institute of Technology, Harbin 150001, China; 2024113235@stu.hit.edu.cn (S.Z.); 2022110806@stu.hit.edu.cn (T.Y.)

**Keywords:** autocollimator, aberrations, assembly deviation, sensitivity of assembly deviation

## Abstract

**Highlights:**

**What are the main findings?**

- Among the various aberrations of a collimator objective, only coma at various orders affects the accuracy of an autocollimator.
- The angle errors introduced by assembly deviation are a systematic shift. This shift is independent of the field of view and is determined solely by the aberrations and the decentration of the collimator objective. It can be expressed in terms of the Sensitivity of Assembly Deviation (SAD) coefficient.

**What are the implications of the main findings?**

- The coma-dominant influence can be used to correct the aberrations of a collimator objective during the design step.
- The Sensitivity of Assembly Deviation coefficient can be utilized to guide the positional and attitudinal adjustments of the collimator objective during the adjustment step.

**Abstract:**

Autocollimators are widely used for sensor calibration. It is crucial to suppress systematic errors and enhance the measurement accuracy of autocollimators. However, the influence mechanism of systematic errors originating from the collimator objective—such as aberrations, and particularly the coupled effect of aberrations and assembly deviation—on measurement accuracy is not well understood. By switching between the perspectives of ray tracing and aberration, we analyzed the influence mechanism of aberrations. The results indicate that only coma aberrations of various orders affect the accuracy. Then we applied nodal wave aberration theory and found that the influence of assembly deviation is a factor only related to the aberration under a certain offset (composed of translational and rotational components). In this work, we defined the sensitivity of assembly deviation as the ratio of this factor and the offset. In the experimental part based on a homemade autocollimator, the maximal errors of yaw angle are decreased from 2.09 arcsecs to 1.41 arcsecs, while the one of pitch angle is decreased from 2.32 arcsecs to 1.63 arcsecs, within a measuring interval of ±500 arcsecs. The sensitivity of assembly deviation of the collimator objective used is 0.004 arcsec per micron, which agrees with the theoretical analysis.

## 1. Introduction

An autocollimator is an important precision instrument for small-angle measurement in industry [1,2], usually working as a transfer measurement device to calibrate other sensors. For example, Mou et al. used an autocollimator with an accuracy of 1 arcsec for in situ calibration of fiber optic gyroscopes, reducing the measurement errors to under 3 arcsec within a full range of 360 degrees [3]. Another example is Arif Sanjid, who used an autocollimator with an accuracy of 0.04 arcsec to calibrate digital levels, reducing the measurement errors under 1.77 arcsec within the measurement range of 1030 arcsec [4]. According to the calibration hierarchy, the measurement accuracy of the calibrator should be higher than the sensors being calibrated. Therefore, reducing the errors of autocollimators is a key issue in the field of small-angle measurement.

**Figure 1:**
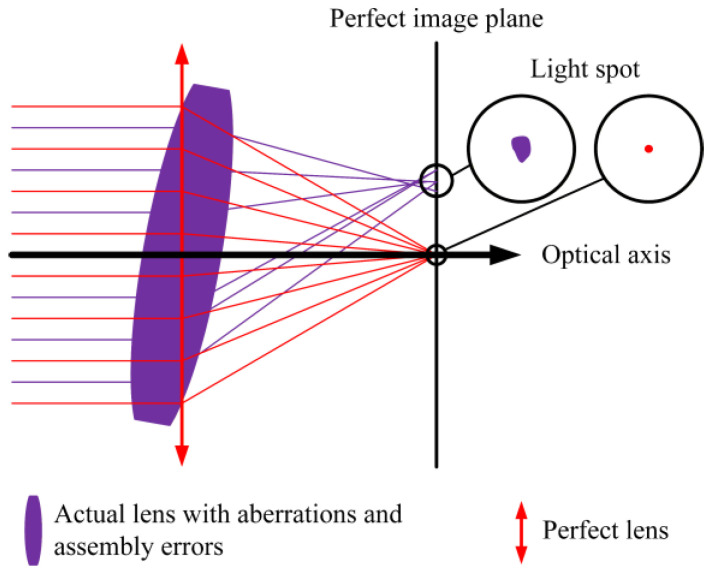
Schematic of image quality of perfect and actual lenses influenced by aberrations and assembly deviation.

Normally, an autocollimator includes a light source, a diaphragm, a beam-splitting prism, a collimator objective, a target mirror, a photodetector, and the corresponding signal processing electronics. Suppressing or eliminating errors is an effective approach to improve the measurement accuracy of an autocollimator. Researchers have proposed several inspiring methods for reducing errors of autocollimators among the abovementioned parts:

- Laser source. Li et al. proposed an algorithm for suppressing the influence of an imperfect Gaussian light source, especially for long working distances. They successfully reduced the errors caused by unbalanced light distribution to one-sixth [5]. Zhu et al. established a ray-tracing model based on the Gaussian formula, which could be used for analyzing the influence of an imperfect dotted light source [6]. The model above treats the lens as an ideal lens without any aberration.
- Diaphragm. Li et al. found that reducing the roughness of the diaphragm could result in improvements in measurement uncertainties [7]. Yu et al. used a diaphragm of array silts to average the influence of the manufacturing errors from each silt [8]. Fuetterer studied the effect of coherence on the imaging effect of slit diaphragms [9]. Lovchy et al. investigated the vignetting features of different slits and proposed a slit-selecting method for different areas of measuring range [10,11]. However, the use of the Diaphragm has also brought about new problems. When multiple slits are used, there will be cases where the object points deviate from the optical axis. The influence of aberrations on autocollimation angle measurement will be more complex.
- Target mirror. The expanded-size beam could reduce the impact of mirror defects on reflected light by the average effect. Dagne et al. designed a mini-autocollimator with an extra beam expander [12]. The systematic errors by the 8 mm beam were reduced to 11.274 arcsec, compared to 22.099 arcsec errors by the 2 mm beam. Eves and Leroux proposed an approach to find the direction of the normal vector of a real mirror with an imperfect surface, which is also proven to be effective in reducing errors [13]. Hiraku Matsukuma et al. investigated autocollimators with rough surfaces [14]. They revealed that the systematic errors induced by the rough surface could be suppressed by selecting a specific wavelength.
- The above research provides effective methods to reduce the impact of mirrors. However, it does not take into account the influence of an imperfect lens. When the impact of the mirror is coupled with the aberration of the lens, the influence on angle measurement becomes more complex.
- Especially, the collimator objective is attributed to the quality of the collimated beam, which has received special attention from researchers. As Figure 1 shows, the assembly deviation will not deteriorate the image quality of a perfect lens without aberration. However, an actual lens with aberrations will produce an irregularly shaped spot, with a deviated centroid. The assembly deviation will further affect the shape of the light spot and the position of the centroid. Therefore, aberrations and assembly deviation are two coupled systematic errors of a collimator objective.
- Collimator objective. Shimizu et al. analyzed the influence on sensitivity caused by spherical aberration of the collimator objective and achieved a high resolution of 1 milli-arcsec [15]. Shi et al. investigated the assembly deviation of the lens by ray tracing. The experiment result shows that a lens offset of 20 μm resulted in a 13.09 arcsec increase in the optical aberration within a measuring range of 1000 arcsec [16]. The above research did not establish an explicit relationship between aberration and angle measurement error.

However, the aberration of the collimator objective is complicated, contributed by five components [17,18] (spherical aberration, coma, astigmatism, field curvature, and distortion). The mechanism of how the aberrations influence the measuring accuracy of the autocollimator has not been theoretically investigated. Additionally, since the assembly deviation determines the position and attitude of the light source and collimator objective, which is coupled with aberrations. There is no method to divide influences by assembly deviation and aberrations, which becomes a barrier on the way to higher-precision autocollimators.

In this work, we focused on the mechanism of systematic errors in the collimator objective on measuring the accuracy of an autocollimator. In the theoretical part, we established the relationship between aberrations and measuring accuracy based on Zernike polynomials and found that only the coma influences. This allows us to pay more attention to the effects of coma when designing a collimator objective for autocollimators. Then nodal aberration theory is used in analyzing the coupling relationship between aberrations and assembly deviation. We found that the influence of assembly deviation is a factor only related to the aberration under a certain offset (composed of translational and rotational components), but independent of the field of view. In this work, we defined the sensitivity of assembly deviation (SAD) as the ratio of this factor and the offset, for a clear and quantized description. In the experimental part based on a homemade autocollimator, the maximal errors of yaw angle are decreased from 2.09 arcsec to 1.41 arcsec, while the one of pitch angle is decreased from 2.32 arcsec to 1.63 arcsec, within a measuring range of ±500 arcsec. The SAD of the collimator objective used is 0.004 arcsec per micron, which agrees with the theoretical analysis.

## 2. Principle and Simulation

### 2.1. Coordinate System Definition

The definitions of all the variables in this chapter are presented in Table 1. Most of the variables are the coordinates at the exit pupil of the optical system or on the image plane. The other variables are the first-order optics parameters related to angle measurement.

**Figure 2:**
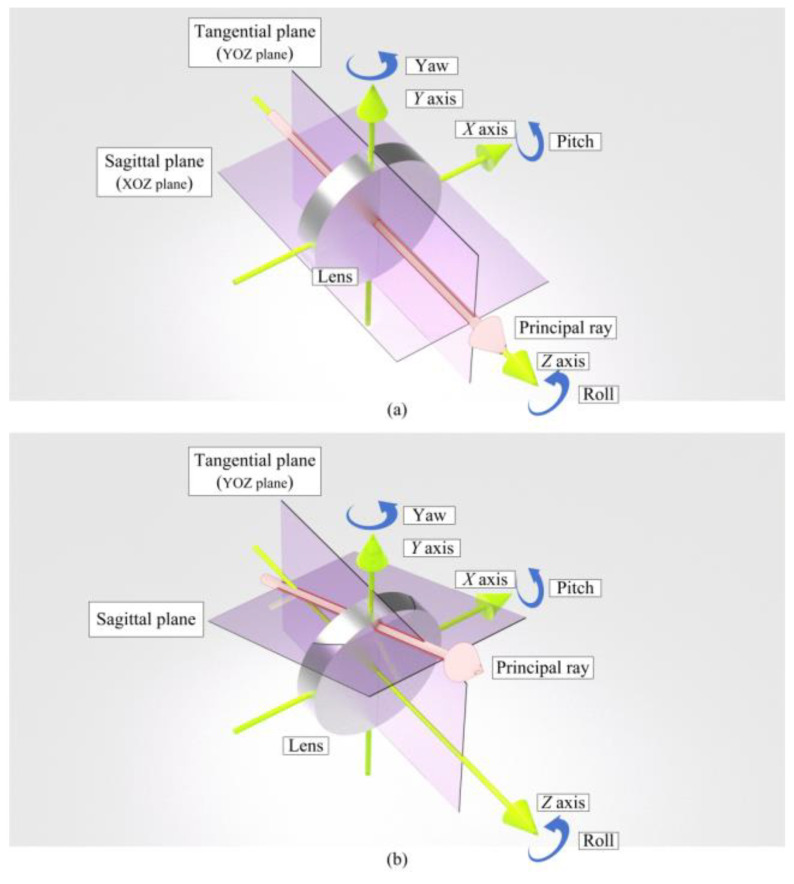
Definition of the planes and angles in a Cartesian coordinate system: (**a**) The principal ray of the beam coincides with the optical axis of the lens. (**b**) The principal ray of the beam has an angle with the optical axis of the lens.

It is necessary to define a coordinate system to clearly explain the directions and assembly deviation. As Figure 2 shows, the optical axis is along the *Z-*axis. The tangential plane is defined as the *YOZ* Plane. As Figure 2a shows, the principal ray of the beam coincides with the optical axis of the lens, and the sagittal plane is defined as the *XOZ* plane. As Figure 2b shows, the principal ray of the beam does not coincide with the optical axis of the lens, and the sagittal plane is defined jointly by the tangential plane and the principal ray of the beam. The rotation angles with respect to *X-*, *Y-*, and *Z*-axes are pitch, yaw, and roll angles, with an anti-clockwise position direction.

Analyzing six degrees of freedom is a complex task. At the beginning, it is better to make some reasonable simplifications and separate the degrees of freedom that we are concerned with. In this work, we regard the collimator objective to be radially symmetrical, ignoring the profile deviation caused by manufacturing and fixture. Then the roll angle could be ignored. In addition, if the lens deviates in the *Z*-axis, the image will be out of focus. The defocusing could be solved by adjusting the camera so that the *Z*-axis deviation could also be ignored.

Then the offset of the collimator objective is the deviation along the *X*- and *Y*-axes. Further, because of the symmetric, the remaining four degrees of freedom could be separated into two interchangeable parts: pitch angle, *X*-axis deviation (and the *XOZ* plane); as well as yaw angle, *Y*-axis deviation (and the *YOZ* plane). Therefore, the symmetric could be used to further simplify the simulation and experiment.

### 2.2. Mechanism of the Aberrations on Autocollimation Angle Measurement

#### 2.2.1. Principle

**Figure 3:**
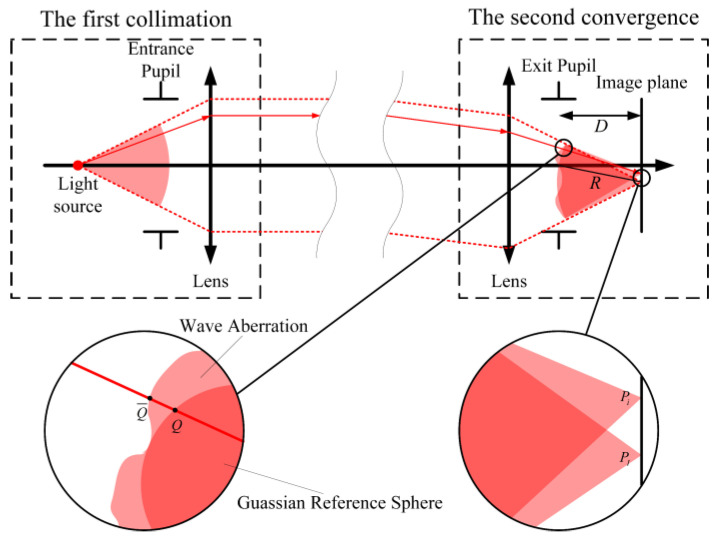
Unfolding schematic of angle measurement in an autocollimator.

As Figure 3 shows, the light passes a collimator objective twice in an autocollimator. The first collimation occurs after the diverging light enters the lens via the entrance pupil. The on-axis collimated beam reflects from the target mirror, becoming an off-axis collimated beam. Then, the second convergence occurs before the collimated beam leaves the lens via the exit pupil. The light spot centroid is deviated from the perfect image spot because of the actual collimator objective, resulting in measurement errors. According to the measurement principle of a dual-axis autocollimator, the errors of the pitch angle *error*_pitch_ and yaw angle *error*_yaw_ are expressed as

(1) $$error_{\mathrm{pitch}}=\frac{Y_{\mathrm{cen}}}{f}-\theta_{\mathrm{pitch}}=\frac{Y_{\mathrm{cen}}-f\times \theta_{\mathrm{pitch}}}{f}=\frac{Y_{\mathrm{cen}}-Y_{I}}{f}$$

(2) $$error_{\mathrm{yaw}}=\frac{X_{\mathrm{cen}}}{f}-\theta_{\mathrm{yaw}}=\frac{X_{\mathrm{cen}}-f\times \theta_{\mathrm{yaw}}}{f}=\frac{X_{\mathrm{cen}}-X_{I}}{f}$$

An autocollimator employs a centroid positioning algorithm to determine the *X*_cen_ and *Y*_cen_ of the spot center. The specific calculation process involves determining the coordinates of each individual ray and performing a weighted average operation on all the rays. We sampled *n* rays in the collimated beam, and given that all rays are equally weighted. Then the total weight equates to the number of rays, *n*. Hence, *X*_cen_ is calculated as the sum of all *X_i_* coordinates divided by *n*, and *Y*_cen_ is correspondingly obtained as the sum of all *Y_i_* coordinates divided by *n*. Furthermore, for all sampling rays, the image height of the perfect image point is identical. Therefore, we multiply both the numerator and denominator of *X*_I_ and *Y*_I_ by *n*. Move *X*_I_ and *Y*_I_ within the summation symbol on the numerator. Then Equations (1) and (2) could be modified as

(3) $$error_{\mathrm{pitch}}=\frac{\frac{\sum_{i=1}^{n}Y_{i}}{n}-Y_{I}}{f}=\frac{\sum_{i=1}^{n}\left(Y_{i}-Y_{I}\right)}{n\times f}$$

(4) $$error_{\mathrm{yaw}}=\frac{\frac{\sum_{i=1}^{n}X_{i}}{n}-X_{I}}{f}=\frac{\sum_{i=1}^{n}\left(X_{i}-X_{I}\right)}{n\times f}$$

Equations (3) and (4) indicate that the measurement errors of an autocollimator are directly related to the *i*-th ray aberrations *Y_i_*-*Y*_I_ and *X_i_*-*X*_I_. As Figure 3 shows, the rays emitted from the dotted light source on the object plane enter the collimator objective through the entrance pupil. Supposing that the plane of exit pupil represents the intersection of the ray and the perfect spherical wavefront, while the point $\bar{Q}$ on the plane of exit pupil is the intersection of the ray and the actual distorted wavefront caused by aberrations. On the image plane, the perfect image of the ray is at the point *P*_I_, while the actual image is at the point *P_i_*. The distance between the point *P*_I_ and the center of the exit pupil is the radius *R* of the perfect wavefront. The point *P_i_* is on the extended line passing through the points *Q* and $\bar{Q}$. Therefore, the difference in wavefront $Q\bar{Q}$ at the exit pupil is related to the ray aberration i-th *P_i_P*_I_, which could be expressed as

(5) $$\frac{\partial \Phi }{\partial Y_{Qi}}=\frac{n_{\mathrm{Image}}}{D}\times (Y_{i}-Y_{I})$$

(6) $$\frac{\partial \Phi }{\partial X_{Qi}}=\frac{n_{\mathrm{Image}}}{D}\times (X_{i}-X_{I})$$

In Equations (5) and (6) as well as throughout the following text, we use the symbol *Φ* to represent the wavefront difference.

Usually, the ray trace and the aberrations are two approaches to the same goal—analyzing the measurement errors of an autocollimator. However, the previous works in the field only follow the ray trace approach, because there is no way to transfer to an aberration view. Based on the above Equations (5) and (6), we established the relationship between the ray trace and the aberrations. The two equations could be regarded as a connection to transfer between the two approaches. Then, the measurement errors expressions related to the first-order optics parameter and wave aberration are established as

(7) $$error_{\mathrm{pitch}}=-\frac{D}{f\times n_{\mathrm{Image}}}\frac{\sum_{i=1}^{n}\left(\frac{\partial \Phi }{\partial Y_{Qi}}\right)}{n}$$

(8) $$error_{\mathrm{yaw}}=-\frac{D}{f\times n_{\mathrm{Image}}}\frac{\sum_{i=1}^{n}\left(\frac{\partial \Phi }{\partial X_{Qi}}\right)}{n}$$

When the number of sampled rays in the collimated beam approaches infinity, the summation can be replaced by a double integral over the exit pupil. The pupil coordinates $\left(X_{Qi},Y_{Qi}\right)$ were transformed into polar coordinates to facilitate the integration. The integral form of the autocollimator errors is given by Equations (9) and (10).

(9) $$error_{\mathrm{pitch}}=-\frac{D}{f\times n_{\mathrm{Image}}}\frac{\int_{0}^{\frac{d_{\mathrm{pupil}}}{2}}\int_{0}^{2\pi }\frac{\partial \Phi }{\partial \rho_{Qi}\sin(\theta_{Qi})}\rho_{Qi}d\theta_{Qi}d\rho_{Qi}}{{\left(\frac{d_{\mathrm{pupil}}}{2}\right)}^{2}\times \pi }$$

(10) $$error_{\mathrm{yaw}}=-\frac{D}{f\times n_{\mathrm{Image}}}\frac{\int_{0}^{\frac{d_{\mathrm{pupil}}}{2}}\int_{0}^{2\pi }\frac{\partial \Phi }{\partial \rho_{Qi}\cos(\theta_{Qi})}\rho_{Qi}d\theta_{Qi}d\rho_{Qi}}{{\left(\frac{d_{\mathrm{pupil}}}{2}\right)}^{2}\times \pi }$$

The wavefront aberration Φ of the collimated beam is expanded using Fringe Zernike polynomials. Upon evaluating the double integral, the resulting relationships between the aberration coefficients and the autocollimator errors are given by Equations (11) and (12). Among them, variables beginning with a *Z* (*Z*_2_, *Z*_3_, *Z*_7_, *Z*_8_, etc.) are all the coefficients of the Fringe Zernike polynomials.

(11) $$error_{\mathrm{pitch}}=-\frac{2\times D}{f\times n_{\mathrm{Image}}\times d_{\mathrm{pupil}}}\left(Z_{3}+Z_{8}+Z_{15}+Z_{24}+Z_{35}\right)$$

(12) $$error_{\mathrm{yaw}}=-\frac{2\times D}{f\times n_{\mathrm{Image}}\times d_{\mathrm{pupil}}}\left(Z_{2}+Z_{7}+Z_{14}+Z_{23}+Z_{34}\right)$$

When vignetting is absent, the integral of the wavefront errors contributions from aberrations like spherical, astigmatism, field curvature, and distortion evaluates to zero over the circular domain of the pupil plane. The angle errors are solely related to coma aberrations of various orders, and all orders of coma contribute equally to these errors. Therefore, the greater the magnitude of the coma coefficient, the more pronounced its influence on the autocollimator measurement. Meanwhile, for a given level of coma, the influence of aberrations on the autocollimator’s accuracy increases with a longer exit pupil distance, a shorter focal length, and a smaller exit pupil diameter.

#### 2.2.2. Simulation

**Figure 4:**
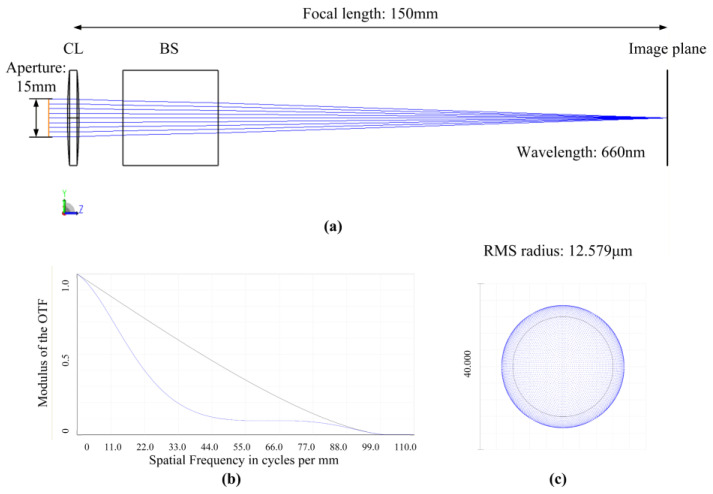
Optical System Layout for Simulation. (**a**) Layout; (**b**) MTF curves; (**c**) Spot diagram.

To validate Equations (11) and (12), an autocollimator system, as shown in Figure 4, was constructed in the optical simulation software ZEMAX (2024R1). Since compact design was not a constraint for the autocollimator, our simulation and experiment employed a single biconvex lens as the collimator objective, rather than a more complex telephoto lens group. Since the analysis above focuses on the wavefront error in the exit pupil. Both aberrations from the lens and the beam splitter can affect the wavefront error in the exit pupil. Therefore, we included the beam splitter (BS) in our simulation. The BS brings astigmatism to the optical system [19].

In the simulation, the rays were set in the following sequence: Light source → Beam Splitter → Collimator Objective → Mirror → Collimator Objective → Beam Splitter → Image Plane. This configuration results in the beam passing through the collimator objective twice—just as an autocollimator does.

**Figure 5:**
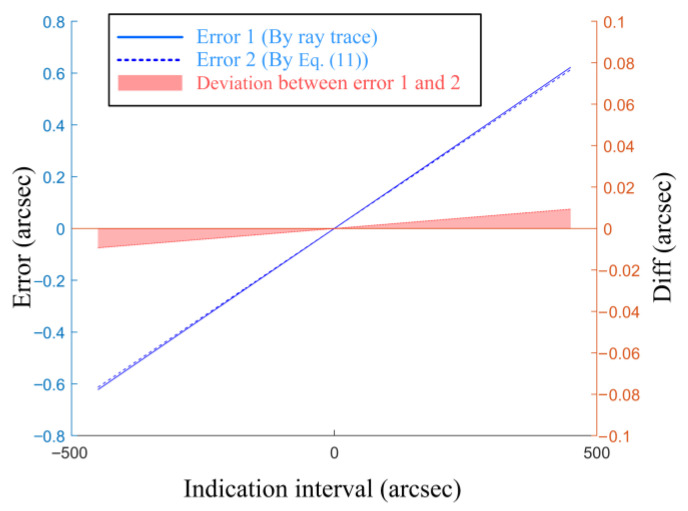
Simulation for the aberration-based mechanism.

A macro was written within ZEMAX. This program calculates the aberration-induced errors in the autocollimator system across the measurement range of −500 arcsec to +500 arcsec, in accordance with Equations (11) and (12). Due to the rotational symmetry of the autocollimator system about the optical axis, the aberrations in the tangential and sagittal planes are identical. Therefore, only the errors in the yaw direction are presented here. As shown in Figure 5, the errors calculated via Equation (11) show no significant discrepancy when compared to the results obtained by the ray trace method. Across the range of −500 arcsec to 500 arcsec, the maximum deviation remains below ±0.01 arcsec and exhibits a linear dependence on the angle. This behavior can most likely be attributed to discrepancies in the numerical precision between the software’s ray trace algorithm and the aberration calculation. This validates the correctness of Equation (11) and confirms that the systematic errors contributed by aberrations are indeed determined by the coma coefficients at various orders and the first-order optics parameters, including the exit pupil distance, exit pupil diameter, and focal length.

The present theory can account for the pattern observed in the simulations and experiments of Ref. [16]: the systematic errors contributed by aberrations exhibit a highly linear relationship with the measurement range. It can be inferred that when the coma of the collimator objective is uncorrected, the magnitude of the primary coma is significantly larger than that of the higher-order coma. Since primary coma *Z*_7_ and *Z*_8_ exhibit linear relationships with the field of view, the resulting errors consequently show a good linearity with respect to the measurement range. If the primary coma of the collimator objective is corrected, and higher-order coma becomes the dominant factor, then the residual errors will exhibit a strongly nonlinear relationship with the measurement range.

### 2.3. Mechanism of Assembly Deviation on Autocollimation Measurement

The definitions of the variables in this chapter are all presented in Table 2. These variables play a significant role in the nodal wave aberration theory.

#### 2.3.1. Principle

From Equations (11) and (12), it can be seen that the angle errors depend on the first-order optics parameter and the coefficients of the coma aberrations at various orders. When the collimator objective suffers from decenter and/or tilt, assembly deviation exists in the optical surfaces. Since first-order optics parameters such as the focal length *f* and exit pupil diameter *D* remain unaffected, the assembly deviation alters the autocollimator’s error distribution by influencing the coma coefficients at various orders. Consequently, understanding how assembly deviation influences aberrations enables the derivation of their impact on autocollimator measurements. Conventional aberration theory is not applicable to the analysis of surfaces with assembly deviation. To address this limitation, this work introduces nodal wave aberration theory for the analysis.

According to nodal wave aberration theory, decenter along the *Y*-axis and tilt about the *X*-axis (pitch errors) affect the aberrations exclusively in the tangential plane; whereas decenter along the *X*-axis and tilt about the *Y*-axis (yaw errors) influence only the sagittal plane aberrations. Given that the collimator objective is rotationally symmetric, resulting in identical aberration distributions in the tangential and sagittal planes, the analysis and discussion in this section are consequently confined to the tangential plane.

**Figure 6:**
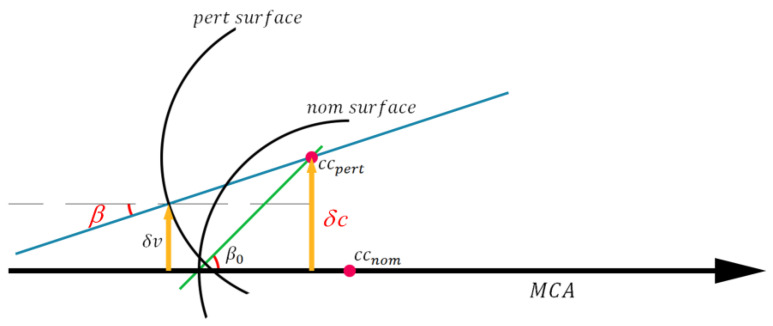
Definition of the parameters in the surface with assembly deviation.

Since the effect of tilt is also to decenter the local axis of the optical surface, we define a certain variable to describe the surface’s position and attitude. As shown in Figure 6, the angle *β*_0_ between the equivalent local axis (the line connecting *cc*_pert_ to the vertex of the optical surface without assembly deviation) and the reference axis (MCA) is defined as the variable that comprehensively captures the tilt and decenter state of the surface with assembly deviation.

(13) $$\beta_{0}=\beta +\frac{\delta v}{r}=\frac{\delta c}{r}$$

For an optical surface with assembly deviation, points *Q*, *Q*′, *E*, *E*′ are no longer collinear; consequently, the system does not possess a conventional optical axis. The line connecting points *Q* and *E* is defined as the Optical Axis Ray (OAR), which serves as the effective optical axis of the optical surface with assembly deviation. Since the OAR is the reference for analyzing aberration changes, the analysis therefore begins by deriving its position.

**Figure 7:**
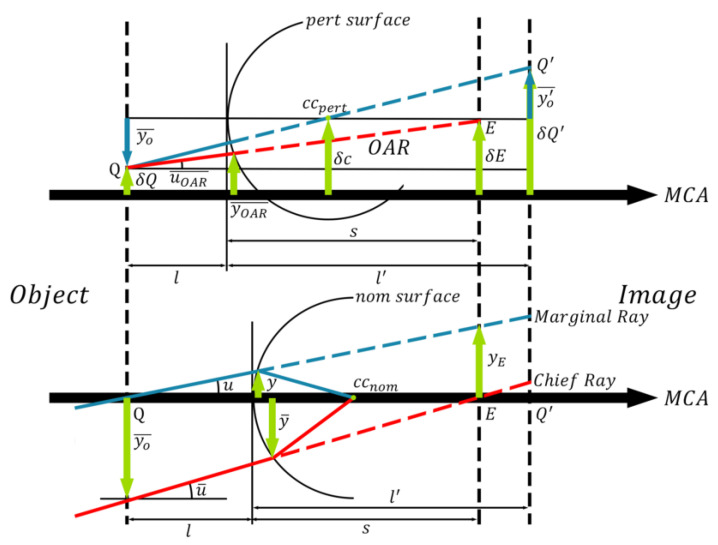
Ray Trace Diagram for Paraxial Rays at Surface with Assembly Deviation.

As shown in Figure 7, determining the paraxial object/image height and pupil radius requires tracing at least two rays: the principal ray from the marginal field of view (MFOV) and the marginal ray from the on-axis field of view (OFOV). The intersection of the principal ray from the MFOV with the optical axis defines the pupil position for this surface. At this location, the height of the marginal ray from the OFOV yields parameters *y_E_*, *y_E_*′. Conversely, the intersection of the marginal ray from the OFOV with the optical axis defines the paraxial image plane position. The height of the principal ray from the MFOV at this position yields parameters $\bar{y_{o}}$, $\bar{{y_{o}}^{\prime }}$.

Although points *Q*, *Q*′ and *cc*_pert_ on the surface with assembly deviation remain collinear, we can geometrically derive that:

(14) $$\frac{\delta Q^{\prime }-\delta c}{l^{\prime }-r}=\frac{\delta c-\delta Q}{r-l}$$

(15) $$\delta Q^{\prime }=-\frac{l^{\prime }-r}{l-r}\times (\delta c-\delta Q)+\delta c$$

Here, $\frac{l^{\prime }-r}{l-r}$ represents the paraxial magnification *m* of the surface, and is equivalent to $\frac{\bar{{y_{o}}^{\prime }}}{\bar{y_{o}}}$. Thus, Equation (15) can be rewritten as:

(16) $$\frac{\delta Q^{\prime }}{\bar{{y_{o}}^{\prime }}}=(\frac{1}{\bar{{y_{o}}^{\prime }}}-\frac{1}{\bar{y_{o}}})\times \delta c+\frac{\delta Q}{\bar{y_{o}}}$$

Thus, given *δQ* of the surface with assembly deviation, and parameters $\bar{y_{o}}$ and $\bar{{y_{o}}^{\prime }}$ of the surface without assembly deviation, *δQ*′ for the surface with assembly deviation can be calculated. Since the image space of the preceding surface is the object space of the subsequent surface, this formula can be propagated surface-by-surface. Similarly, the method for propagating the pupil deviation surface-by-surface is given by Equation (17).

(17) $$\frac{\delta E^{\prime }}{{y_{E}}^{\prime }}=(\frac{1}{{y_{E}}^{\prime }}-\frac{1}{y_{E}})\times \delta c+\frac{\delta E}{y_{E}}$$

With the solution for *δQ*, *δQ*′, *δE*, *δE*′ obtained, and given that the optical axis ray is defined by its passage through both the object space center *Q* and the entrance pupil center *E* of the surface with assembly deviation, the angle between the OAR and the reference axis can be determined using Equation (18).

(18) $$\bar{u_{\mathrm{OAR}}}=\frac{\delta E-\delta Q}{s-l}$$

It follows that $\frac{1}{s-l}$ is equivalent to $-\frac{\bar{u}}{\bar{y_{o}}}$. Given that $\bar{u}$ is the angle between the principal ray from the MFOV and the optical axis, Equation (18) can therefore be rewritten as Equation (19):

(19) $$\bar{u_{\mathrm{OAR}}}=-\frac{\bar{u}}{\bar{y_{o}}}\times (\delta E-\delta Q)$$

From the definition of the Lagrange-Helmholtz invariant, the following equivalence holds in a surface with assembly deviation:

(20) $$-ny_{E}\bar{u}=n\bar{y_{o}}u$$

where u is the angle between the marginal ray from OFOV and the optical axis. Substituting Equation (20) into Equation (19) yields:

(21) $$\bar{u_{\mathrm{OAR}}}=\frac{u}{y_{E}}\times \delta E+\frac{\bar{u}}{\bar{y_{o}}}\times \delta Q$$

Given the known value of $\bar{u_{\mathrm{OAR}}}$, the parameter $\bar{y_{\mathrm{OAR}}}$ can be derived:

(22) $$\bar{y_{\mathrm{OAR}}}=\delta Q-l\times \bar{u_{\mathrm{OAR}}}$$

Here, y is defined as the distance to the optical axis. It is measured from the intersection point of the marginal ray (from OFOV) with the surface that has no assembly deviation, and *l* is equivalent to $-\frac{y}{u}$. By incorporating the relationship between $\bar{u_{\mathrm{OAR}}}$ and *l*, the expression for $\bar{y_{\mathrm{OAR}}}$ can be expanded as follows:

(23) $$\bar{y_{\mathrm{OAR}}}=\delta Q+\frac{y}{u}\times (\frac{u}{y_{E}}\times \delta E+\frac{\bar{u}}{\bar{y_{o}}}\times \delta Q)$$

(24) $$\bar{y_{\mathrm{OAR}}}=\frac{y}{y_{E}}\times \delta E+(\frac{\bar{y_{o}}+\frac{y\times \bar{u}}{u}}{\bar{y_{o}}})\times \delta Q$$

In Equations (23) and (24), the parameter $\bar{y_{o}}+\frac{y\times \bar{u}}{u}$ can be transformed into $\bar{y_{o}}-l\times \bar{u}$. Moreover, since $\bar{y_{o}}-l\times \bar{u}$ is equivalent to the distance $\bar{y}$. Here, $\bar{y}$ is defined as the distance from the optical axis to the intersection point of the principal ray (from the MFOV) with the surface that has no assembly deviation. The expression for $\bar{y_{\mathrm{OAR}}}$ can be simplified as follows:

(25) $$\bar{y_{\mathrm{OAR}}}=\frac{y}{y_{E}}\times \delta E+\frac{\bar{y}}{\bar{y_{o}}}\times \delta Q$$

The optical axis ray for each surface with assembly deviation can be calculated using Equations (19) and (25). The optical axis of the collimator objective, with tilt and decenter, is then formed by these OARs concatenated end-to-end.

**Figure 8:**
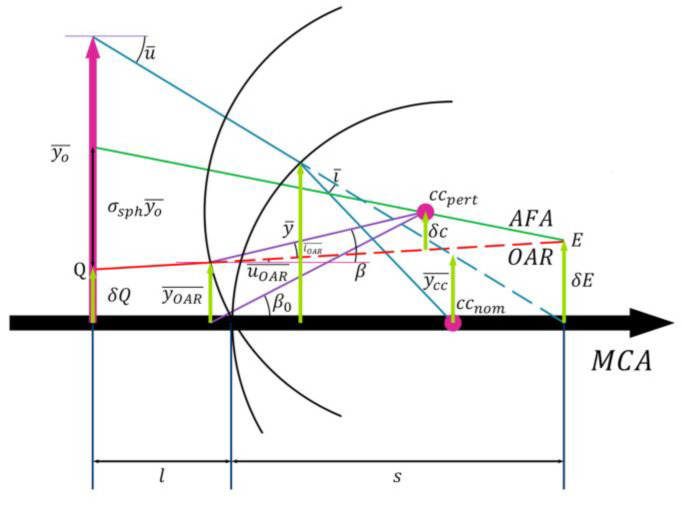
Vector Diagram of Aberration Field Shift for a Surface with Assembly Deviation.

However, the aberration field axis (AFA) of a surface without assembly deviation coincides with the MCA, whereas for a surface with assembly deviation, the AFA does not coincide with the OAR. As shown in Figure 8, the decenter of the aberration field center relative to the MCA at the object plane is defined as $\sigma_{\mathrm{sph}}$, and the distance between the principal ray from the MFOV and the center of curvature of the surface without assembly deviation is defined as $\bar{y_{cc}}$.

(26) $$\frac{s-r}{r-l}=\frac{\delta c}{\sigma_{\mathrm{sph}}\bar{y_{o}}}=\frac{\bar{y_{cc}}}{\bar{y_{o}}}$$

(27) $$\sigma_{\mathrm{sph}}=\frac{\delta c}{\bar{y_{cc}}}$$

Here, $\bar{i}$ and $\bar{i_{\mathrm{OAR}}}$ are the angles of incidence for the principal ray from the MFOV and the optical axis ray, respectively. $\bar{y_{cc}}$ can be expressed as $r\times \bar{i}$, and *δc* as $-r\times \bar{i_{\mathrm{OAR}}}$. Substituting the above parameters into Equation (27) yields:

(28) $$\sigma_{\mathrm{sph}}=\frac{\delta c}{\bar{y_{cc}}}=-\frac{r\times \bar{i_{\mathrm{OAR}}}}{r\times \bar{i}}=-\frac{\bar{i_{\mathrm{OAR}}}}{\bar{i}}$$

Based on the angle relationships depicted in Figure 8, the expressions for $\bar{i_{OAR}}$, $\bar{i}$ can be derived:

(29) $$\bar{i_{\mathrm{OAR}}}=\bar{u_{\mathrm{OAR}}}-\beta =\bar{u_{\mathrm{OAR}}}-(\beta_{0}-\frac{\bar{y_{\mathrm{OAR}}}}{r})$$

(30) $$\bar{i}=\bar{u}+\frac{\bar{y}}{r}$$

And substituted Equations (29) and (30) into (28):

(31) $$\sigma_{\mathrm{sph}}=-\frac{\bar{i_{\mathrm{OAR}}}}{\bar{i}}=-\frac{\bar{u_{\mathrm{OAR}}}-(\beta_{0}-\frac{\bar{y_{\mathrm{OAR}}}}{r})}{\bar{u}+\frac{\bar{y}}{r}}$$

If we define the coma of the optical surface i without assembly deviation is $\Phi_{i}$, then its change induced by tilt and/or decenter is $\sigma_{\mathrm{sph}}\times \Phi_{i}$. The total coma change for the entire optical system is given by:

(32) $$\sum_{i=1}^{n}\sigma_{\mathrm{sph}}\times \Phi_{i}=\sum_{i=1}^{n}-\frac{\bar{u_{\mathrm{OAR}}}-(\beta_{0}-\frac{\bar{y_{\mathrm{OAR}}}}{r})}{\bar{u}+\frac{\bar{y}}{r}}\times \Phi_{i}$$

As indicated by Equation (32), the decenter and tilt of the collimator objective do not introduce new aberrations, but merely shift the center of the original aberration field away from the optical axis, thereby resulting in a uniform change in aberrations across the entire field of view. The magnitude of this change is independent of the field of view, and is solely determined by the aberrations of the collimator objective without assembly deviation along with decenter and tilt. Thus, assembly deviation introduces a field-independent shift in the systematic angle errors caused by aberrations.

#### 2.3.2. Simulation

We established the optical configuration of an autocollimator in ZEMAX for validating Equation (32). Tilt deteriorates the aberrations of the collimator objective by introducing an effective decenter, and its effect is equivalent to that of pure decenter. Moreover, the aberration distributions are identical in the *YOZ* and *XOZ* planes, and the influence of assembly deviation on these two planes is independent. Therefore, the effect of decenter on the autocollimator measurement is simulated solely for the yaw direction.

**Figure 9:**
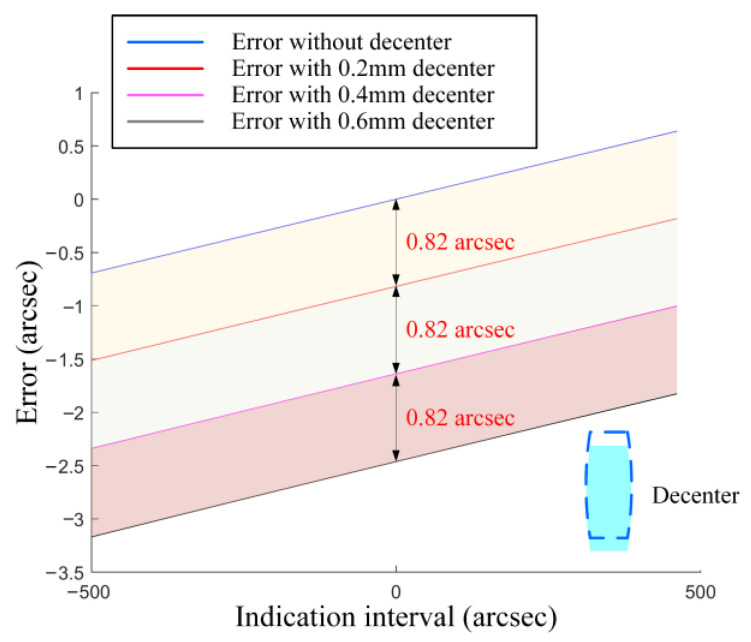
Simulation of the Impact Mechanism of Assembly Deviation.

As shown in Figure 9, the variation in the angle errors shows a linear dependence on the radial decenter of the collimator objective along the *X*-axis. This demonstrates that the systematic errors introduced by assembly deviation depend solely on the aberrations and the magnitude of the decenter of the collimator objective, thus constituting a field-independent quantity. Clearly, this quantity reflects the sensitivity of the collimator objective to assembly deviation. Therefore, we define the ratio of this quantity to the decenter value as the SAD. We performed simulations under four lens conditions: no decenter, and decenters of 0.2 mm, 0.4 mm, and 0.6 mm. Four corresponding sets of data were collected. As shown in the figure, a consistent error shift of 0.82 arcsec is observed between each adjacent data set. Therefore, the SAD Coefficient is calculated to be 0.004 arcsec/μm.

## 3. Experiment

### 3.1. Verification of the Aberration-Based Mechanism

**Figure 10:**
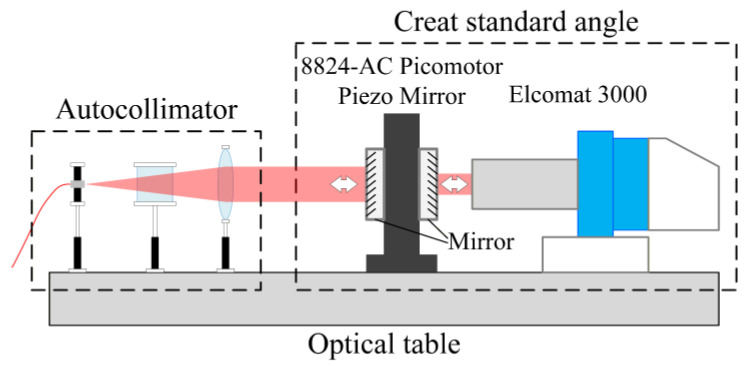
Experimental Setup Schematic for Aberration Mechanism Verification.

**Figure 11:**
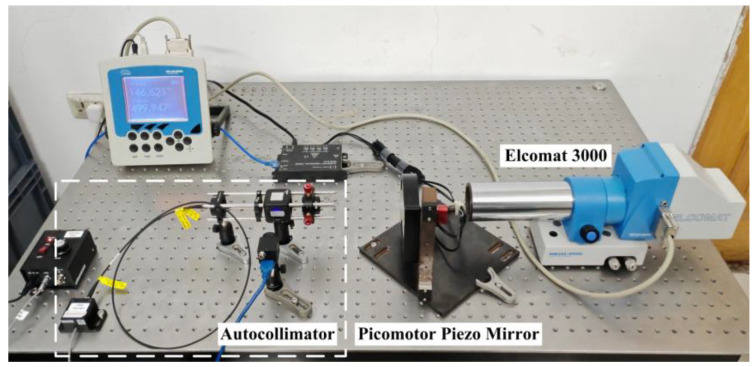
Experimental Setup Photograph for Aberration Mechanism Verification.

An experimental autocollimator setup, identical to the simulation system in Figure 4, was constructed to validate the findings in Section 2.1 regarding the mechanism of how aberrations influence the autocollimator measurement. The collimator objective employs a biconvex lens (Model: MBCX10615-A, Lbtek, Shenzhen, China; focal length: 150 mm; diameter: 25.4 mm; material: N-BK7; radius of curvature: 154.0 mm), and the photodetector is a CMOS area camera (Model: BFS-U3-32S4-C, Flir, Thousand Oaks, CA, USA; pixel size: 3.45 μm; resolution: 4000 × 3000; gain range: 0–47 dB). The light source employs an LED (Model: LFM660, Changchun New Industries Optoelectronics Technology, Changchun, China; wavelength: 660 nm). As shown in Figure 10, the standard angle positioning system comprises an angle-generating unit (8824-AC tilting stage from Newport, Irvine, CA, USA) and a standard autocollimator (MÖLLER-WEDEL OPTICAL, Wedel, Germany). A photograph of the experimental setup is presented in Figure 11.

**Figure 12:**
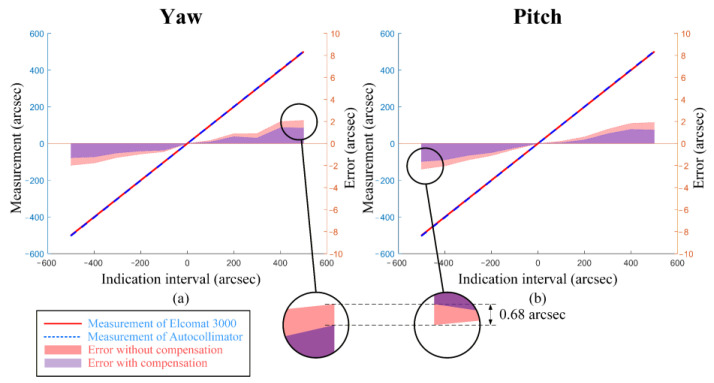
Experimental Verification Results of the Aberration Influence Mechanism: (**a**) Verification Results in the yaw direction. (**b**) Verification Results in the pitch direction.

**Figure 13:**
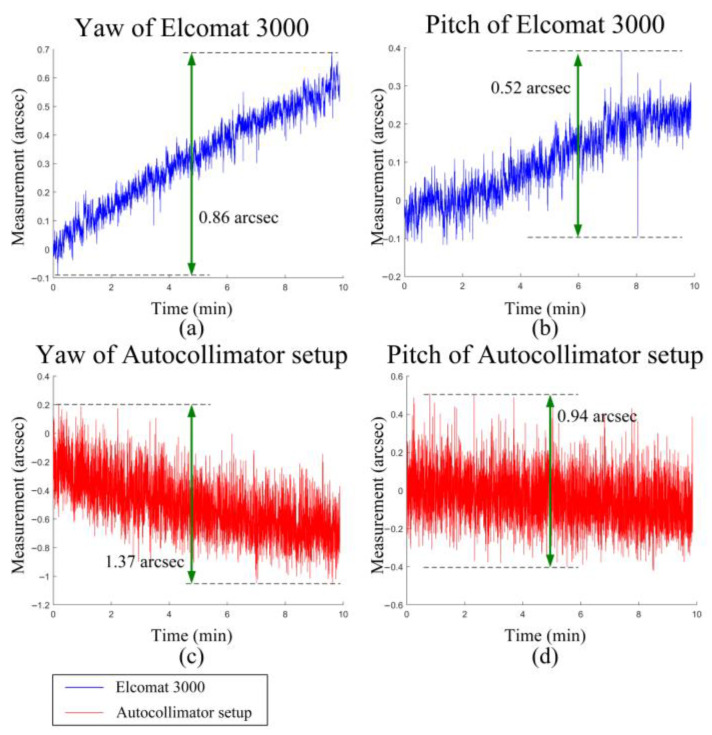
Stability Test Results of the Autocollimation System and the Elcomat 3000: (**a**) Stability test results of Elcomat 3000 (yaw). (**b**) Stability test results of Elcomat 3000 (pitch). (**c**) Stability test results of Autocollimator setup (yaw). (**d**) Stability test results of Autocollimator setup (pitch).

The angle errors of the setup in both the yaw and pitch directions are shown in Figure 12. The measurement results were then corrected for the aberration-induced errors calculated from Equations (11) and (12) in Section 2.2.2 (as previously shown in Figure 5). The results demonstrate that after compensation, across the full measurement range of ±500 arcsec, the maximum errors in the yaw direction were reduced from 2.09 arcsec to 1.41 arcsec, and in the pitch direction from 2.32 arcsec to 1.63 arcsec. As shown in Figure 13, limited by the environmental control and vibration isolation conditions of the laboratory, the short-term (10 min) stability of the autocollimator setup and the Elcomat 3000 (MÖLLER-WEDEL OPTICAL) was 1.37 arcsec and 0.86 arcsec, respectively. The differing drift directions observed in the figure arise from the differing coordinate system conventions used by the Elcomat 3000 and the autocollimator setup. This indicates that random errors introduced by the experimental environment are a significant source of the residual errors, thereby validating the effectiveness of the proposed methodology.

### 3.2. Verification of the Assembly Deviation Mechanism

**Figure 14:**
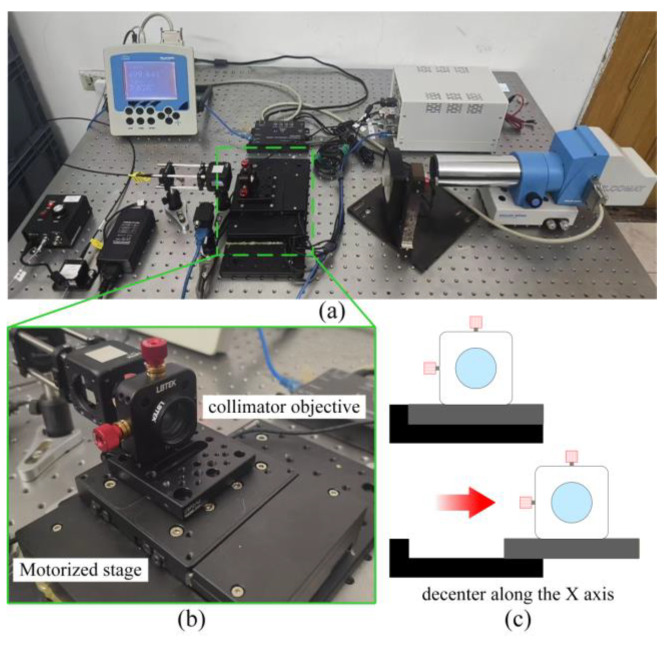
Photograph of the Setup for Verifying the Mechanism of Assembly Deviation: (**a**) Overall diagram of the experimental setup. (**b**) A close-up picture of the collimating objective is fixed on a motorized stage. The motorized stage enables the collimating objective to undergo displacements of the order of micrometers. (**c**) A diagram showing that the collimating objective decenter along the X axis is moving with the motorized stage.

As shown in Figure 14, the lens was mounted on a piezoelectric translation and tilt stage from United Optics (model number: 526658, United Optics, Shanghai, China). Featuring a built-in grating scale, the stage allows for the accurate generation of microscopic motions (displacement resolution of 1 micrometer), thus facilitating precise radial decenter of the collimator objective along the *X*-axis.

**Figure 15:**
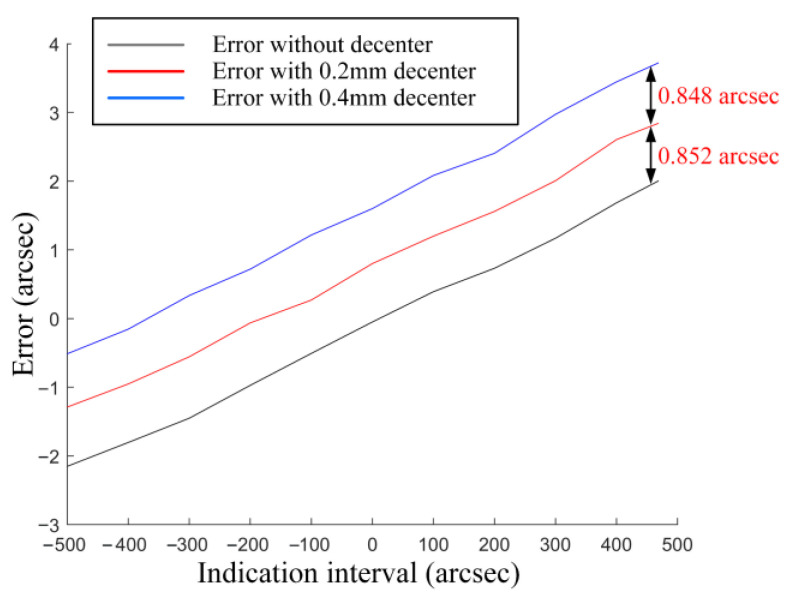
Experimental Verification Results of the Impact Mechanism of Assembly Deviation.

As illustrated in Figure 15, when the collimator objective was continuously displaced along the *X*-axis, the systematic errors demonstrated a consistent variation. The calculated SAD Coefficient is 0.0043 arcsec/μm. The experimentally obtained sensitivity coefficient exhibits a difference of 0.0003 arcsec/μm compared to the simulation results from Section 2.2.2. This minor deviation confirms the validity of the research findings presented in Section 2.2. The observed discrepancy can be attributed not only to the laboratory environmental conditions illustrated in Figure 13, but also to the performance specifications of the translation and tip/tilt stage.

## 4. Discussion

In the analysis above, we have found that among the aberration components, only coma significantly affects the measurement accuracy of an autocollimator. Compensating for first-order coma can effectively improve accuracy. In this Section, we mainly discuss the issues beyond the coma: the vignetting, the wavelength, and the type of lens.

**Figure 16:**
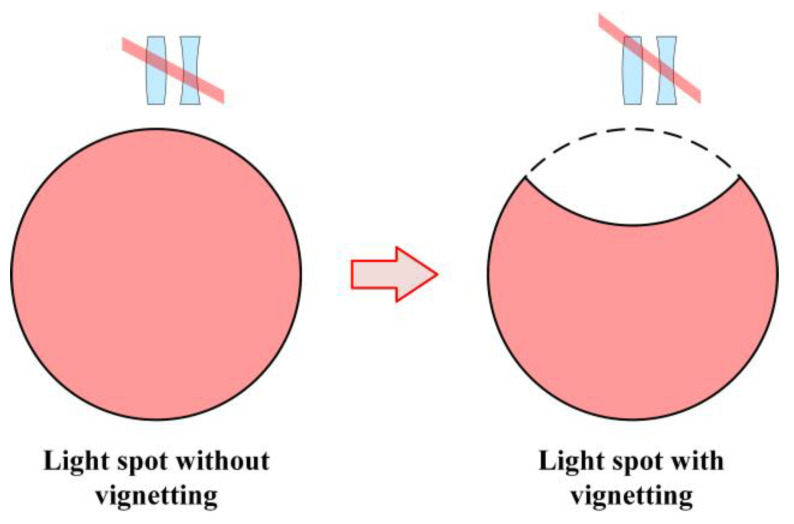
The shape of the light spot in the absence of vignetting and in the presence of vignetting.

**Figure 17:**
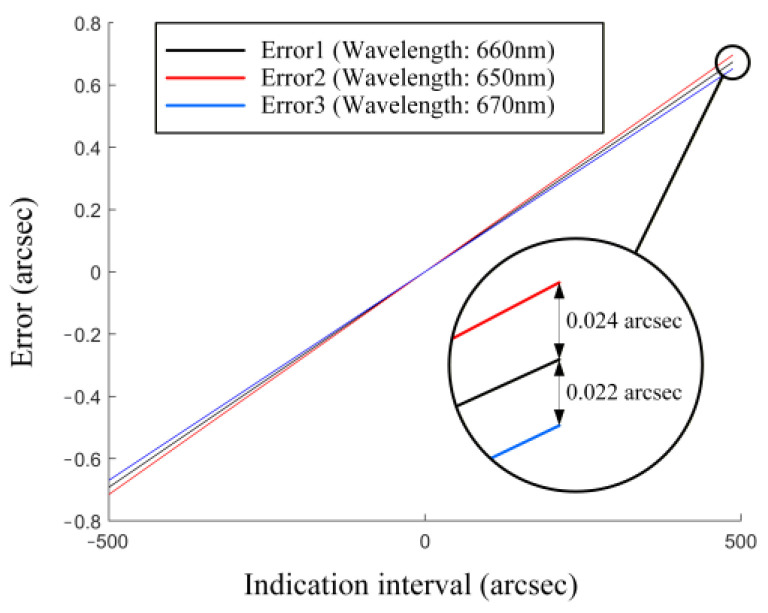
Simulation for the aberration-based errors at different wavelengths.

- About the vignetting. As shown in Figure 16, when the optical system has vignetting, the shape of the light spot loses its symmetry. At this point, the idealized circular pupil integration cannot be achieved. This will result in each Zernike coefficient no longer being 0 or an integer multiple of π. In this case, the relationship between the aberration and the angle error is no longer as simple as shown in Equations (11) and (12). However, when the vignetting significantly influences, the collimator objective is not available for an autocollimator anymore. Therefore, when designing the autocollimator, one should avoid the occurrence of vignetting. And that is why the coma is mainly investigated in this work.

- About the center wavelength. The center working wavelength used for all the simulations above was 660 nm, consistent with the nominal wavelength of the light source used in the experiments. Obviously, the real center wavelength is not exactly 660 nm. When the center wavelength alters, the linear relationship presented in this article is still applicable, but only differs in value. Figure 17 shows the simulation results at different wavelengths. Since the wavelength range of the used light source is 660 ± 10 nm, we selected 650 nm and 670 nm for further investigation. It could be seen that the angle measurement still exhibits good linearity within the measurement range. The only difference is that when the wavelength changes, the slope of the error line undergoes a slight alteration. That is because the values of the aberration coefficients will change under different wavelengths.
- About the chromatic aberration. Some autocollimators equip two light sources with significantly different wavelengths to implement measurement of three rotation angles simultaneously [20]. Longitudinal and lateral chromatic aberrations would cause different wavelengths to focus at different positions. Therefore, the influence of chromatic aberrations on angle measurement should be taken into account. An achromatic objective is an effective solution for such autocollimator designs.

- About the type of lens. Both the simulations and experiments in this paper investigate an optical system with biconvex lenses. During the derivation of the formulas in this paper, the optical system was treated as a black box. Discussions on aberrations and assembly errors were all conducted at the exit pupil. Therefore, the conclusions drawn in this paper remain valid when the optical lens is a meniscus lens or any other type. In addition, it can be known from Equations (11) and (12) that some optical system parameters have an impact on the measurement error. For instance, when other variables remain constant, the higher the optical power of the lens, the greater the angle error. This can help us select or design a suitable collimator objective for the autocollimator.

## 5. Conclusions

In this work, we focus on the mechanism of how systematic errors in collimator objectives influence the angle results of an autocollimator. In the theoretical part, we derived the relationship between the aberrations in the collimator objective and the systematic component of angle errors, which is expressed by a formula. Specifically, we have found that only the coma counts for the measurement accuracy, while the influence of other aberrations could be eliminated by the centroid calculation algorithm. The systematic errors could be reduced by mitigating the first-order coma first, and then the higher-order coma contributes.

The coupling relationship of the two key contributors to systematic errors, assembly deviation and aberrations, was established by employing the nodal wave aberration theory. The influence of systematic errors caused by assembly deviation could be expressed by a coefficient. This coefficient, called the sensitivity of assembly deviation (SAD), depends on aberrations and the decenter of the collimator objective, while it is independent of the measured angle. Its value is directly proportional to the amount of decenter.

We compensated for the aberrations in a homemade autocollimator. Experimental results of the ±500 arcsec measurements demonstrate that the maximum errors of the yaw angle were reduced from 2.09 arcsec to 1.41 arcsec; the maximum errors of the pitch angle decreased from 2.32 arcsec to 1.63 arcsec. Further, we tested the SAD-coefficient value of the used collimator objective is 0.004 arcsec/μm. The experimental results (0.0043 arcsec/μm) are consistent with the simulation data. It reveals that a 1-μm decentration of the collimator objective leads to a 0.0043-arcsec error. We believe the above experimental results could prove the validity of the theoretical work.

In the future, we plan to develop an autocollimator considering the above relationships. On the one hand, coma at various orders will be suppressed during the optical design of the collimator objective. On the other hand, the SAD coefficient of the collimator objective will be tested and used to indicate a precise assembly. We hope this work could contribute to the field of autocollimator by explaining the relationships between the collimator objectives and the systematic errors.

## Figures and Tables

**Table 1:** All variables appearing in the full text and their corresponding definitions.

Variables Name	Definitions of Variables
$\theta_{\mathrm{pitch}}$	Pitch angle, the angle subtended between the principal ray and the optical axis in the *YOZ* plane
$\theta_{\mathrm{yaw}}$	Yaw angle, the angle subtended between the principal ray and the optical axis in the *XOZ* plane
f	Focal length of the collimator objective
$Y_{\mathrm{cen}}$	The image height of the light spot centroid for the off-axis parallel beam’s image spot in the *YOZ* plane
$X_{\mathrm{cen}}$	The image height of the light spot centroid for the off-axis parallel beam’s image spot in the *XOZ* plane
$Y_{I}$	The image height of the perfect image point in the *YOZ* plane
$X_{I}$	The image height of the perfect image point in the *XOZ* plane
$Y_{i}$	The image height of the *i*-th ray from the off-axis parallel beam in the *YOZ* plane
$X_{i}$	The image height of the *i*-th ray from the off-axis parallel beam in the *XOZ* plane
$Y_{Qi}$	The coordinate in the tangential direction of the intersection point between the *i*-th ray from the off-axis parallel beam and the actual wavefront
$X_{Qi}$	The coordinate in the sagittal direction of the intersection point between the *i*-th ray from the off-axis parallel beam and the actual wavefront
$n_{\mathrm{Image}}$	The refractive index in the image space of the optical system
D	The exit pupil distance of the optical system
$d_{\mathrm{pupil}}$	The exit pupil diameter of the optical system
Φ	The wavefront error in the exit pupil

**Table 2:** All variables appearing in the full text and their corresponding definitions.

Variable Name	Definitions of Variables
$cc_{\mathrm{nom}}$	The center of curvature of optical surface without assembly deviation lies on the reference axis
$cc_{\mathrm{pert}}$	The center of curvature of optical surface with assembly deviation lies off the reference axis
β	The surface inclination of optical surface with assembly deviation
δc	The center deviation of the optical surface with assembly deviation
δv	The vertex deviation of the optical surface with assembly deviation from the reference axis
Q	Object space center of the optical surface
$Q^{\prime }$	Image space center of the optical surface
E	Center of the entrance pupil for the optical surface
$E^{\prime }$	Center of the exit pupil for the optical surface
δQ	The deviation of the object space center of the optical surface with assembly deviation from the reference axis
$\delta Q^{\prime }$	The deviation of the image space center of the optical surface with assembly deviation from the reference axis
δE	The deviation of the entrance pupil center of the optical surface with assembly deviation from the reference axis
$\delta E^{\prime }$	The deviation of the exit pupil center of the optical surface with assembly deviation from the reference axis
$\bar{y_{o}}$	Paraxial object height of the optical surface
$\bar{{y_{o}}^{\prime }}$	Paraxial image height of the optical surface
$y_{E}$	Entrance pupil radius of the optical surface
${y_{E}}^{\prime }$	Exit pupil radius of the optical surface
l	The object intersection distance of the optical surface
s	Entrance pupil distance of the optical surface
r	Radius of curvature of the optical surface
$\bar{u_{\mathrm{OAR}}}$	The angle subtended between the optical axis ray and the reference axis
$\bar{y_{\mathrm{OAR}}}$	The deviation of the intersection point between the optical axis ray and the optical surface with assembly deviation from the reference axis

## Data Availability

The raw data supporting the conclusions of this article will be made available by the authors on request.

## Abbreviations

The following abbreviations are used in this manuscript:

SAD	Sensitivity of Assembly Deviation
MCA	Mechanical coordinate axis
OAR	Optical Axis Ray
MFOV	Marginal field of view
OFOV	On-axis field of view
AFA	Aberration field axis
MTF	Modulation Transfer Function
BS	Beam splitter
CL	Collimator lens
RMS	Root mean square
